# Intra-host SARS-CoV-2 diversity in immunocompromised people living with HIV provides insight into the evolutionary trajectory of SARS-CoV-2

**DOI:** 10.1128/jvi.00780-25

**Published:** 2025-09-05

**Authors:** R. Joseph, G. Marais, I. Iranzadeh, A. Alisoltani, D. Hardie, M.-A. Davies, A. Heekes, N. Chetty, V. Timmerman, N.-Y. Hsiao, C. Williamson

**Affiliations:** 1 Division of Medical Virology, Institute of Infectious Disease and Molecular Medicine, University of Cape Town, Cape Town, South Africa; 2 Department of Medical Microbiology, University of Cape Town, Cape Town, South Africa; 3 National Health Laboratory Service, Groote Schuur Hospital, Cape Town, South Africa; 4 Department of Microbiology-Immunology, Feinberg School of Medicine, Northwestern University 12244, Chicago, Illinois, USA; 5 Department of Medicine, Division of Infectious Diseases, Feinberg School of Medicine, Northwestern University 12244, Chicago, Illinois, USA; 6 Center for Pathogen Genomics and Microbial Evolution, Havey Institute for Global Health, Feinberg School of Medicine, Northwestern University 12244, Chicago, Illinois, USA; 7 Department of Medical Virology, University of Cape Town, Cape Town, South Africa; 8 Centre for Infectious Disease Epidemiology and Research, School of Public Health, University of Cape Town, Cape Town, South Africa; 9 Health Intelligence Directorate, Western Cape Government Health and Wellness, Cape Town, South Africa; The Ohio State University, Columbus, Ohio, USA

**Keywords:** SARS-CoV-2, COVID-19, immunocompromised, intra-host, evolution

## Abstract

Ongoing viral evolution in immunocompromised individuals with persistent infection may facilitate the evolution of SARS-CoV-2 and emergence of variants of concern (VOC). This study was conducted in the Western Cape Province of South Africa where the HIV prevalence is around 8%, with limited information on the frequency of persistent SARS-CoV-2 infection, the pattern of evolution in these individuals, and if these variants contribute to the diversity of circulating viruses. This study investigated 75 individuals with two or more SARS-CoV-2 diagnoses at least one month apart. Of the 75, 13 were people living with Human Immunodeficiency Virus (PLWH) of which three were immunocompromised, 23 were HIV-negative, and the status of the remaining 39 was unknown. SARS-CoV-2 full-length genome sequence analysis identified 72 as reinfections with a distinct variant and 3 as persistent infections with B.1.1 (20B), B.1.1.459 (20B), and B.1.351 (20H) for 7, 4, and 3 months, respectively. All persistent infections were in severely immunocompromised PLWH with CD4+ T cell count below 30 cells/µL. We identified the emergence of uncommon mutations (global prevalence <0.01%) in SARS-CoV-2, together with permanent and/or transient non-lineage defining mutations with immune escape potential particularly in Spike (141-144del; 241-244del; D215G; E484K; Q498R; P681R; A701V). Some of these mutations were found in later VOCs including Omicron lineages (L18F; 141-144del; D215G; E484K; Q498R; P681R; A701V). Longitudinal viral sequences from these persistent infections provided insights into the evolutionary trajectory of SARS-CoV-2 and are suggestive of convergent evolution and host adaptation.

**IMPORTANCE:**

Unlike other respiratory viruses, SARS-CoV-2 has not yet established a seasonal pattern. Thus, resurgence and the emergence of novel variants including VOCs remain a concern. Ongoing SARS-CoV-2 replication in immunocompromised individuals may serve as reservoirs that could facilitate the emergence of mutations conferring transient and/or long-lasting immune escape potential and seed future outbreaks. Two of the five variants, Beta variant and Omicron variant, were first described in Southern Africa—a region with one of the highest rates of HIV infection globally. Targeted genomic surveillance in immunocompromised individuals including PLWH will provide insight into the evolutionary trajectory of SARS-CoV-2 and inform vaccine design that may help to circumvent resurgence.

## INTRODUCTION

The rapid diversification and dissemination of novel severe acute respiratory syndrome coronavirus 2 (SARS-CoV-2) variants drove consecutive waves of infection worldwide. The emergence of variants with the ability to evade both natural immunities achieved through previous infections, and vaccine-induced immunity, threatens the efficacies of mitigation measures.

The primary hypothesis for the emergence of divergent variants is the accelerated rate of accumulation of mutations during infections in immunocompromised individuals for extended periods (1). Ongoing SARS-CoV-2 replication in different classes of immunocompromised individuals led to the identification of mutations that were detected and/or emerged in subsequent variants of concern (VOCs) (2–7) that pose increased risk to public health. These include adaptive viral changes that confer enhanced transmissibility, pathogenicity, and immune evasion.

Two of the five VOCs, Beta and Omicron, were first described in Southern Africa (8, 9)—a region with the highest rate of HIV infection globally. In South Africa, an estimated 8.45 million people are living with HIV as of 2022 (https://www.statssa.gov.za/publications/P0302/MidYear2022.pdf). However, the frequency of persistent SARS-CoV-2 infections and how the evolutionary dynamics in these cases contribute toward local viral diversity are poorly understood. Advanced HIV infection characterized by low CD4+ T cell counts of less than 200 cells/µL is a cause of compromised immunity. SARS-CoV-2 infection in immunocompromised PLWH led to rapid evolution and an increase in intra-host Spike diversity throughout the infection compared to healthy PLWH and people who are HIV negative. The increase in intra-host viral diversity in immunocompromised PLWH was apparent soon after COVID-19 symptom onset, with increased viral diversity serving as a predictor of persistent infection (10). Although COVID-19 has become endemic, there is not a clear pattern of outbreaks that occur during specific times of the year, and understanding further viral diversification and resurgence remains important. Thus, there is a need for community-based surveillance studies to ascertain the frequency of persistent infections and to characterize intra-host diversity in cases of persistence to gain further insight into the evolutionary trajectory of SARS-CoV-2.

This work describes the viral intra-host evolutionary dynamics in PLWH who are persistently infected with SARS-CoV-2 in the Western Cape Province of South Africa during the COVID-19 pandemic. We observed fluctuations in mutation frequency overtime, with the emergence of non-lineage defining Spike immune escape mutations, some of which were detected in subsequent VOCs and mutations with a low global prevalence at the time of detection. This supports the hypothesis that persistent SARS-CoV-2 infection in immunocompromised PLWH facilitates viral adaptation, diversification, and the emergence of novel variants.

## MATERIALS AND METHODS

### Study design, samples, case series, and ethics

This is a retrospective observational study that made residual SARS-CoV-2 diagnostic specimens (nasopharyngeal swabs, saliva, and tracheal aspirates). Samples were received from the National Health Laboratory Services in Cape Town as part of the SARS-CoV-2 genomic surveillance program (University of Cape Town Human Research Ethics Committee Reference R021/2020 and 383/202). Consecutive SARS-CoV-2 positive specimens originating from individuals with two or more laboratory- confirmed tests with at least one month between the initial and subsequent test were considered for this study.

### Whole-genome sequencing

Total viral ribonucleic acid (RNA) was extracted from residual SARS-CoV-2 positive specimens according to the chemagic Viral300 360 H96 drying prefilling VD200309.che protocol on the ChemagicTM 360 automated system (PerkinElmer, Inc, Waltham, MA). For libraries generated using the Illumina Nextera Flex DNA Library Prep kit, complementary deoxyribonucleic acid was synthesized using Superscript IV First Strand synthesis system (Life Technologies, Carlsbad, CA) and random hexamer primers. SARS-CoV-2 whole-genome amplification was achieved through multiplex polymerase chain reaction according to the ARTIC V.3/V.4/V4.1 protocol using primers designed on Primal Scheme (http://primal.zibraproject.org/) to generate 400 bp amplicons with an overlap of 70 bp that spans the 30 kb genome of SARS-CoV-2. Amplicons were purified using Ampure XP purification beads (Beckman Coulter, High Wycombe, UK) and quantified using the Qubit dsDNA High Sensitivity assay on the Qubit instrument (Life Technologies, Carlsbad, CA). Indexed paired-end libraries were prepared on the Hamilton Next Generation StarLet (Hamilton Company). Libraries were also prepared using the Illumina COVIDSeq kit according to the manufacturer’s protocol. DNA libraries were normalized to 4 nM concentration and pooled and denatured with 0.2 N sodium acetate. A 4 pM sample library was spiked with 1% PhiX Control v.3 adaptor-ligated library. Libraries were sequenced on a 500-cycle v.2 MiSeq Reagent Kit on the Illumina MiSeq instrument (Illumina, Inc., San Diego, CA).

### Genome assembly, alignment, and phylogenetic analyses

Sequence quality assessment and assembly were performed using Exatype (https://sars-cov-2.exatype.com/). Multiple sequence alignment was conducted using the MAFFT algorithm to align the sequences, allowing for comparative analysis (11). Phylogenetic tree construction was then carried out using IQtree 2, a state-of-the-art maximum likelihood phylogenetic inference tool, to infer the evolutionary relationships among the sequences (http://www.iqtree.org/). To incorporate temporal information into the phylogenetic analysis, timed scaled trees were constructed using treetime, enabling the visualization of evolutionary dynamics over time (https://academic.oup.com/ve/article/4/1/vex042/4794731?login=false). Finally, visualization of the phylogenetic trees was achieved using the ggplot and ggtree R packages, facilitating the interpretation and presentation of the results. All genomes were analyzed relative to a global reference data set of contemporaneous genomes from South Africa and using a custom build of the SARS-CoV-2 NextStrain (https://github.com/nextstrain/ncov). Major SARS-CoV-2 clades are assigned to sequences using both the Nextclade server v.0.9 (https://clades.nextstrain.org/) and Phylogenetic Assignment Of Named Global Outbreak Lineages (PANGOLIN). Lineage assignment was confirmed using maximum likelihood phylogenetic analyses.

### Variant analyses

Variant Call Format (VCF) files were retrieved from National Center for Biotechnology Information Sequence Read Archives (https://www.ncbi.nlm.nih.gov/sra). Briefly, VCF files were generated by inter-linking bioinformatics pipelines that align raw sequence reads to the reference genome SARS-CoV-2 Wuhan-Hu-1 isolate (NC_045512, Version: NC_045512.2) and identified and filtered variants for genotyping. These were performed using GenotypeGVFs, HaplotypeCaller, bcftools, and SnpEff. Polymorphic sites and deletions were confirmed and read depth established by visual inspection using Genomics Viewer version 2.14.1 (12). Read depth was determined by counting the number of reads mapped to a given site containing the alternate allele/deletion relative to the total number of reads mapped to the site (variant reads/ total read depth). ViralVar (http://viralvar.org/; 13) was used to visualize variant kinetics using input data obtained from GISAID (https://gisaid.org/).

### Structural analyses

The trimeric structure of the spike protein was visualized using customized R script. Mutations identified in the sequence analysis were mapped onto the spike protein structure (PDB ID: 7a93).

## RESULTS

### Identification of SARS-CoV-2 cases with two positive tests at least 1 month apart

**Fig 1:**
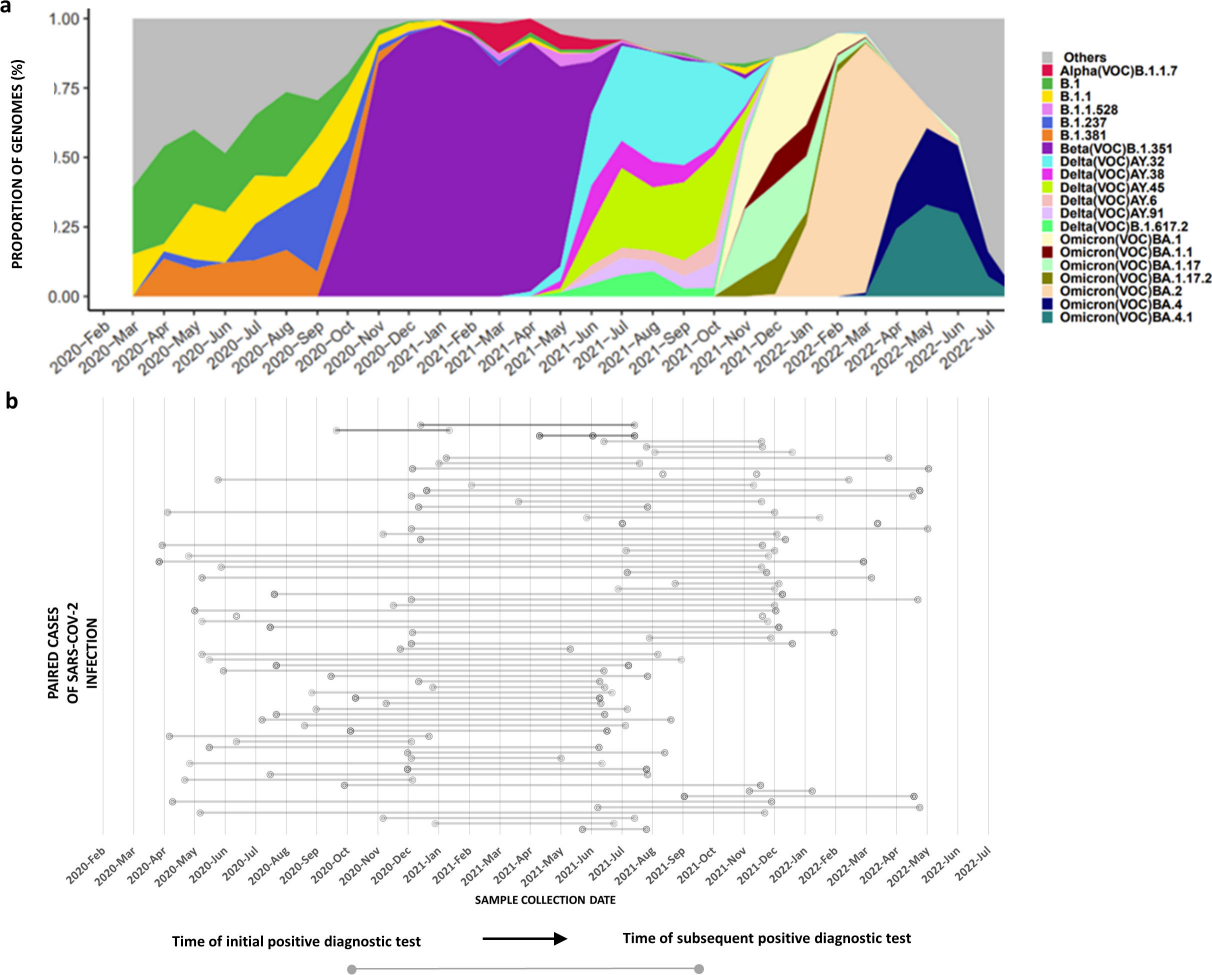
Distribution of SARS-CoV-2 variants in South Africa and collection dates of paired SARS-CoV-2 cases of reinfection and persistence. (**a**) The proportion of SARS-CoV-2 genomes generated by the UCT-NHLS genomic surveillance hub between 2020 and 2022 and the corresponding variant classification. (**b**) The distribution of paired SARS-CoV-2 cases detected. Paired cases are represented by dots joined by solid lines, with the time of the first positive diagnostic test represented by the dot on the left end of the solid line, and the time of subsequent positive diagnostic tests represented by dots to the right end of the solid line. Darker gray lines represent cases of persistence.

To identify persistent infections, we screened 5,938 routine diagnostic SARS-CoV-2 positive specimens collected between April 2020 and May 2022 and identified 75 individuals (1.3%) who had positive diagnostics tests at least 1 month apart, with a mean number of days between tests of 324 days (range 41–692) (Fig. 1b, 48 females and 27 males). During this time, there were changes in the dominant circulating viral variants responsible for the five major waves of infection nationally (ancestral, Beta, Delta, and Omicron variants, Fig. 1a) (14). The average cycle threshold (CT) value for the first test was 23 (range 15–35, 47 individuals CT <25) and 25 (range 13–37) for subsequent tests. Of the 75 individuals, 13 were PLWH, 23 were HIV negative, and 39 were of unknown status. Of the 13 PLWH, three were severely immunocompromised with CD4+ T cell counts <200 cells/µL, while three had CD4+ T cell counts between 200 and 500 cells/µL, four had CD4+ T cell counts >500 cells/µL, and three had no CD4+ T cell count recorded.

### Phylogenetic identification of cases of SARS-CoV-2 persistence

**Fig 2:**
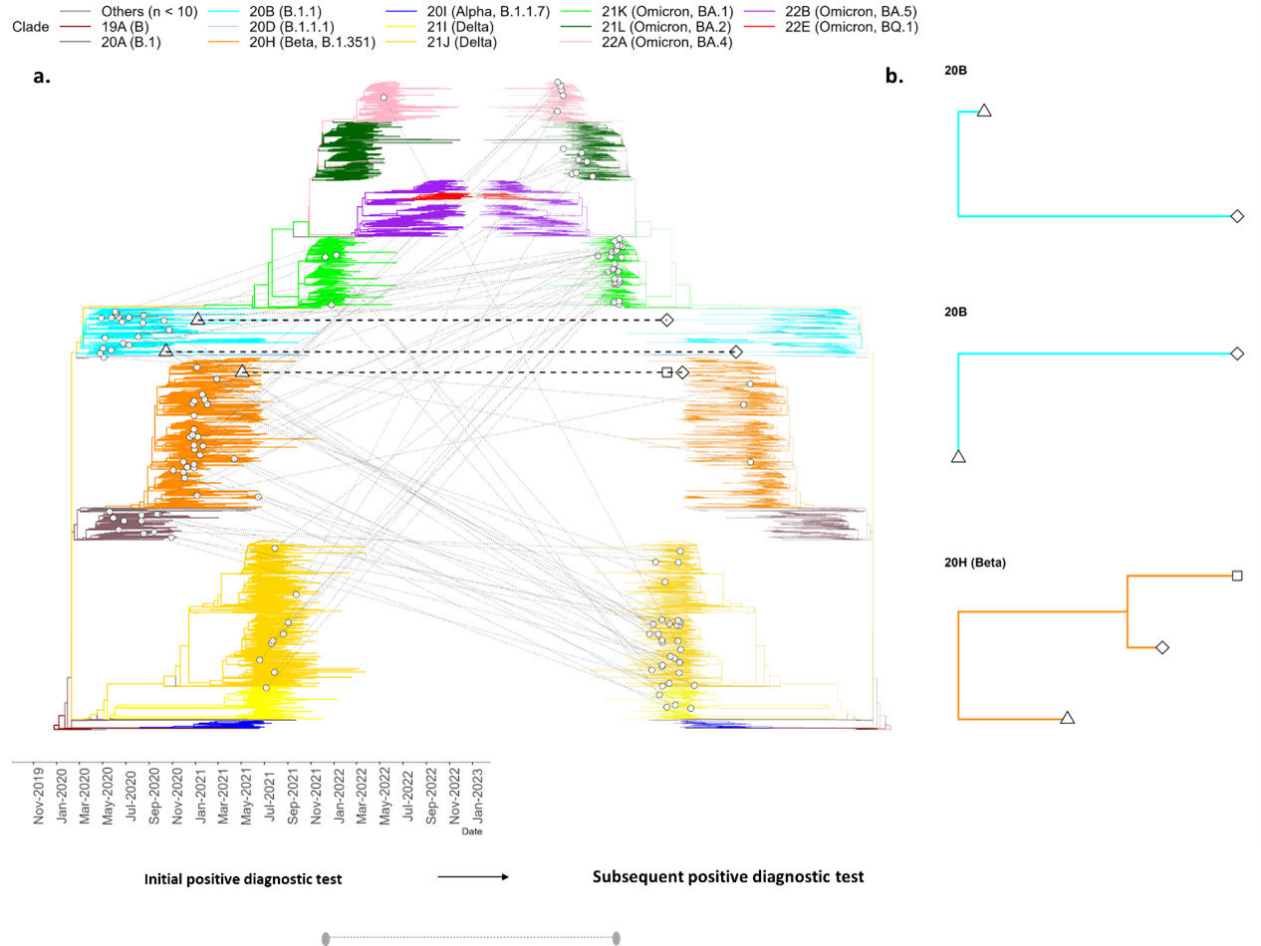
Phylogenetic analysis of SARS-CoV-2 reinfection and persistent infection. (**a**) Mirrored phylogenetic tree showing the placement of the sequence from the first SARS-CoV-2 diagnostic test on the left-hand side and placement of sequences from subsequent SARS-CoV-2 diagnostic tests on the right-hand side. Sequences originating from a single individual are represented by white circles and are joined by light gray lines for reinfections, white triangles/squares joined by black staggered lines for persistent infections. The time of the first positive diagnostic test represented by a shape (triangles/circles) on the left end of the solid/staggered lines, and the time of subsequent positive diagnostic tests represented by a shape (squares/circles) on the right end of the solid/staggered lines. (**b**) Sub-trees showing the phylogenetic relationship between consecutive sequences for cases of SARS-CoV-2 persistence. Sequences are shown relative to 5,548 SARS-CoV-2 from the Western Cape Province, South Africa, between January 2020 and December 2022.

Phylogenetic analyses confirmed that 72 of the 75 individuals were infected with genetically distinct lineages at the time of the initial and subsequent diagnostic tests, indicating reinfection. In the reinfections, the infecting variant matched the dominant variants that were circulating at the time of diagnosis (Fig. 1 and 2). The cases of reinfection included 10 PLWH, 7 with CD4+ T cell counts greater than 200 cells/µL, and 3 with no data on CD4+ T cell counts. Of the remaining 62, 23 were HIV negative, and 39 did not know their HIV status. In contrast, three individuals, followed for between 93 and 210 days, were classified as persistent infection and had the same lineage at the time of initial diagnosis and subsequent diagnostic test. In all cases, the lineage matched the prevailing circulating lineage at the time of the first test and not the subsequent tests (Table 1, Fig. 1 and 2). Case 1 was infected with B.1.351, and cases 2 and 3 were infected with B.1.1.459 and B.1.1, respectively (Fig. 2 and Table 1). These three cases of persistent infections were from severely immunocompromised individuals living with HIV with their CD4+ T cell counts below 30 cells/µL around the time of the SARS-CoV-2 diagnostic tests and were reported to be on antiretroviral treatment (Table 1).

**TABLE 1:** Infection history of immunocompromised individuals persistently infected with SARS-CoV-2

Case	Collection date (yr/mo/day)	Days between positive tests	Lineage (clade)	CD4+ T cell count (cells/µL) (days between CD4+ T cell count and COVID-19 test)	GISAID accession^*a*^	SRR accession
Case 1	2021/05/03	0	B.1.351 (Beta, 20H)	28 (26)	EPI_ISL_2375993	SRR25423973
	2021/06/24	51	B.1.351 (Beta, 20H)	28 (137)	EPI_ISL_3506423	SRR25461190
	2021/08/04	93	B.1.351 (Beta, 20H)	28 (60)	EPI_ISL_11970996	SRR25461171
Case 2	2020/10/15	0	B.1.1.459 (20B)	2 (0)	EPI_ISL_1040670	SRR25419503
	2021/02/03	111	B.1.1.459 (20B)	3 (0)	EPI_ISL_1817752	SRR25423989
Case 3	2021/01/06	0	B.1.1 (20B)	8 (134)	EPI_ISL_3957791	SRR25460872
	2021/08/04	210	B.1.1 (20B)	8 (188)	EPI_ISL_3957821	SRR25461170

^*a*^ Sequence data available via GISAID EPI_SET: EPI_SET_250722mcDOI: https://doi.org/10.55876/gis8.250722mc*.*

### Intra-host evolution of SARS-CoV-2 in immunocompromised PLWH

**Fig 3:**
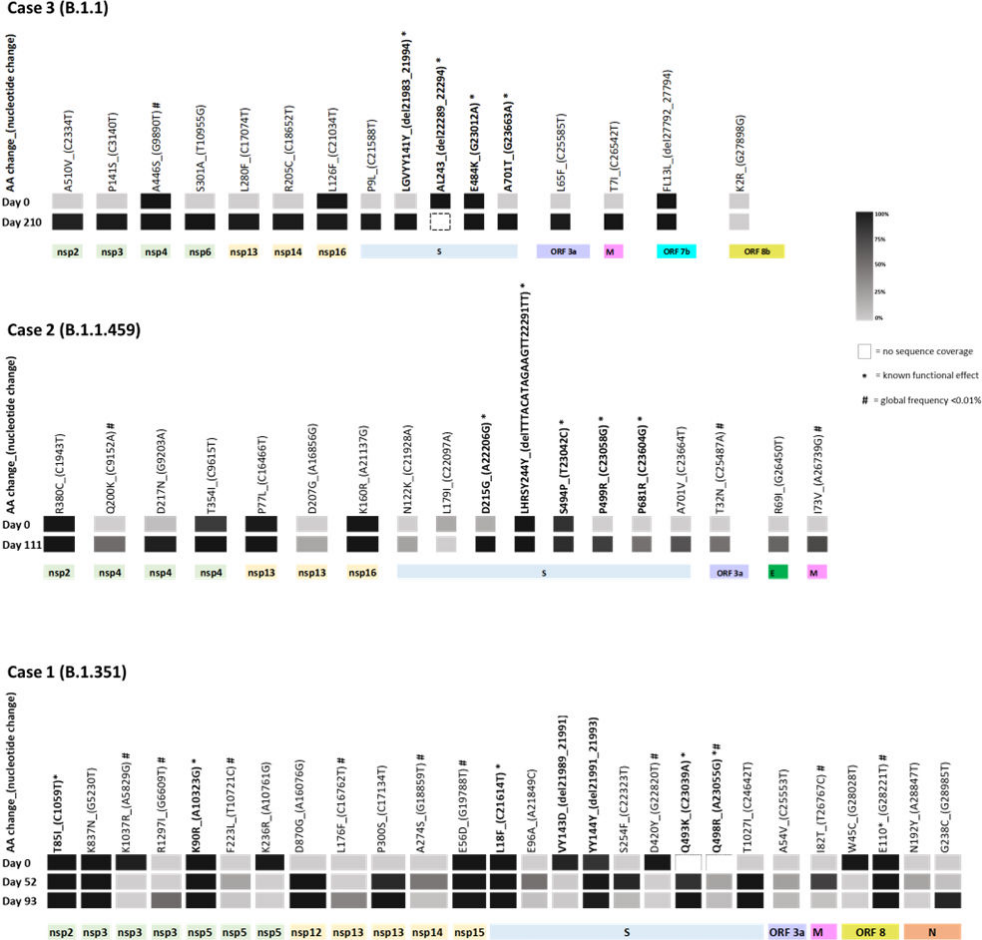
Intra-host molecular evolution of SARS-CoV-2 persistence. Heat map showing variant read depth (% frequency) of non-lineage defining non-synonymous mutations following SARS-CoV-2 persistence in three immunocomprised individuals living with HIV infected with Beta variant (20H) for 93 days, B.1.1.459 (20B) for 111 days, and B.1.1 (20B) for 210 days. nsp, non-structural protein (open read frame 1a, green and open read frame 1b, yellow); S, spike; E, envelope; M, membrane; N, nucleocapsid.

**Fig 4:**
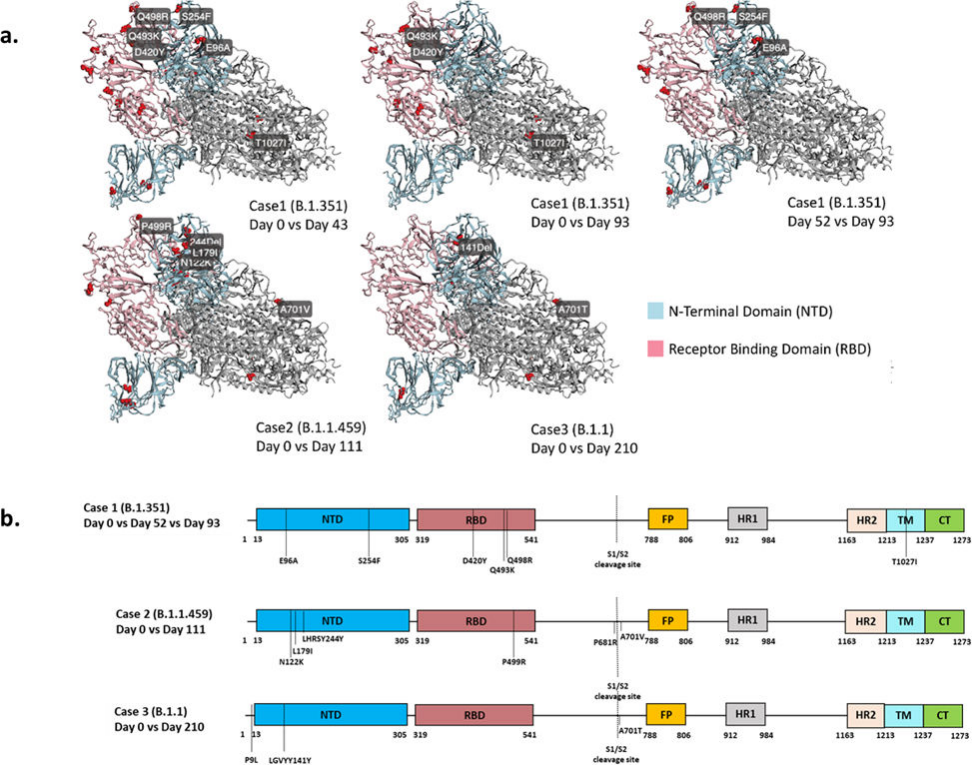
SARS-CoV-2 Spike protein mutations observed during persistent infections. (**a**) Three-dimensional representation of the trimeric structure of the SARS-CoV-2 spike protein, with *de novo* mutations identified in three cases, compares the initial infection stages to subsequent days, highlighting evolutionary changes over time. Red spheres on the structure indicate mutated residues, with mutations labeled on only one chain. Mutations on unresolved regions of the protein structure were not represented including P681R. The N-terminal domain (NTD) and receptor binding domain (RBD) are colored in light blue and pink, respectively, while the rest of the structure is gray. (**b**) Linear representation of Spike indicating amino acid changes and deletions detected at different timepoints during each case of persistent infection. Pink represents the receptor-binding domain and blue represents the N-terminal domain. NTD, N-terminal domain; RBD, receptor-binding domain; S1/S2, S1/S2 cleavage site; S2, S2′ cleavage site; FP, fusion peptide; HR1, heptad repeat 1; HR2, heptad repeat 2; TM, transmembrane anchor; CT, cytoplasmic tail.

Case 1 was a 51-year-old male infected with B.1.351 (Beta variant, 20H), detected in the first sample in May 2021 (Fig. 1b and 2a orange), at a time when there was a switch in circulating variants when the Beta variant was being replaced by the Delta variant (8). This individual remained infected with Beta variant at day 52 and day 93 after the initial test (Fig. 2b). Sequence analysis identified 27 sites with non-lineage defining mutations, comprising of both permanent and transient mutations. The permanent mutations make up 26% of mutations in ORF 1a (7 of 27 sites), 19% in ORF 1b (5 of 27 sites), and 33% in Spike (9 of 27 sites) (Fig. 3 and Table 2). Of these, seven mutations were observed at high read depth frequencies (84%–100%) in sequences from days 0-93 (ORF 1a: T85I, K837N, K90R; ORF 1b: E56D; S: L18F, YY144Ydel; ORF 8: E110*/stop codon). The emergence of additional Spike mutations (Q493K, Q498R, and T1027I) is evident in sequences from day 52 and day 93 (Fig. 3 and Table 2). While Q498R appears to be transient, coverage in these regions is poor in sequence from day 0 (Fig. 3; Fig. S1); thus, one cannot exclude the possibility that these mutations were present in sequences from day 1. One of the fixed mutations in ORF 8, E110*/ stop codon was detected at low global frequency <0.01% (15, 16). Most of the observed Spike mutations were identified in variants both preceding (Alpha variant) and following Beta variant (Gamma and Omicron variants) and are indicative of convergent evolution (Table 1). The structural analysis highlights newly emerged Spike mutations between the different timepoints with the mutations confined to the N-terminal domain (E96A and S254F), the receptor binding domain (D420Y, Q493K, and Q498R), and the transmembrane anchor (T1027I) (Fig. 4).

**TABLE 2:** Functional implication of non-lineage defining Spike mutations identified in immunocompromised individuals persistently infected with SARS-CoV-2^*a*^

	Non-lineage specific mutation	Functional implication	Experimental method(s)	Detected in SARS-CoV-2 variants	Reference(s)
Case 1: infecting variant B.1.351 (Beta, 20H)	L18F	Neutralization resistance	Neutralization assays with convalescent-phase sera and vaccine-elicited antibodies, structural analyses, and binding assays	Beta, Gamma	(17, 18)
	VY143Ddel	Neutralization resistance	Neutralization assays and structural analyses	Alpha, Omicron	(17, 18)
	YY144Ydel	Neutralization resistance	Neutralization assays and structural analyses	Alpha, Omicron	(17, 18)
	Q493K	Reduced Ab binding and neutralization resistance	Neutralization assays and pseudotyped lentivirus assays with serum from vaccinated, boosted donors, and therapeutic monoclonal antibodies, structural analyses		(19)
	Q498R	Increased ACE2-binding affinity particularly in combination with N501Y	Binding affinity assays and structural analyses	Omicron	(20)
Case 2: infecting variant B.1.1.459 (20B)	D215G	Increase cell-to cell fusion	Cell culture assays and structural analyses	Beta	(21)
	LHRSY244Ydel			NTD deletions 241–243 in Beta	
	S494P	Neutralization resistance	Neutralization assays with convalescent-phase sera and vaccine-elicited antibodies		(22, 23)
	P499R	Neutralization resistance	Pseudovirus neutralization assays*		(24)
	P681R	Alters viral tropism by enhancing S1/S2 cleavage in human airway epithelial cells	Replication competition assay, cleavage assay, and reversion mutation assay**	Delta, Kappa, P681H in Alpha and Omicron	(25)
	A701V			Beta	
Case 3: infecting variant B.1.1 (20B)	LGVYY141Ydel	Resistance to NTD-binding neutralizing mAbs	Neutralization assays and structural analyses	NTD deletions 141–146 in Alpha and Omicron	(17, 18)
	AL243del	Resistance to NTD-binding neutralizing mAbs	Neutralization assays and structural analyses	NTD deletions 241–243 in Beta	(18)
	E484K	Increased ACE2-binding affinity, resistance to convalescent sera and NTD-binding neutralizing mAbs	Pseudovirus neutralization and binding affinity assays	Beta, Gamma, E484A in Omicron	(23, 26)
	A701T			701V in Beta	

^*a*^ Vesicular stomatitis virus and/or lentiviral pseudovirus systems (Wuhan-Hu-1 backbone) were used to investigated functional implication. *D614G and B.1.1.529 variant backbones used and **Alpha and Delta variant backbones used. N-terminal domain.

Case 2 was a 24-year-old female infected with ancestral B.1.1.459 (20B), first detected in October 2020 (Fig. 1b and 2a) during the inter-wave period transitioning from wave dominant by ancestral variants to Beta variant-driven wave of infection (Fig. 1a) (14), and remained infected with B.1.1.459 (20B) for 111 days. Forty-four percent of the mutations were in Spike (8 of 18 sites), 22% in ORF 1a (4 of 18 sites), and 17% in ORF 1b (3 of 18 sites) (Fig. 3). The sequences were characterized by the emergence of non-lineage defining mutations that are present in sequences from samples at day 0 and day 111 (ORF 1a: R380C, T354I; ORF 1b: P77L, K160R; **S**: LHRSY244Ydel, S494P) (Fig. 4) high read depth frequencies (80%–100%). There was a notable shift in the emergence of mutations that were either undetected in the sequence from the day 0 sample or present at low read depth frequencies (<10%), but increased in frequency at day 111 (>50%) (ORF 1a: Q200K, D217N; S: D215G, P499R, P681R, A701V; ORF 3a: T32N; E: R69I; M: I73V) with only several observed at a frequency of 70%–100% (ORF 1a: D217N; S: D215G, P499R**;** M: I73V) (Fig. 3). Of note the Spike mutations were mapped to the N-terminal domain (LHRSY244Ydel), the receptor binding domain (P499R), and upstream and downstream of the furin cleavage site (P681R and A701V; Fig. 4).

Case 3 is a 42-year-old female initially identified as infected with B.1.1 (20B) in January 2021 (Fig. 1b and 2a) also during the Beta variant-driven wave of infections (Fig. 1a) (14) when B.1.1 prevalence was low (Fig. 1a). Of the 25 sites identified with permanent non-lineage defining mutations, 31% were in Spike (5 of 16 sites), 25% in ORF 1 a (4 of 15 sites), and 19% in ORF 1b (5 of 16 sites) (Fig. 3). Of note is the E484K mutation at day 0 and day 210 (Fig. 3). Case 3 is also typified by a general shift in the mutational pattern with most of the novel mutations undetected at day 0 (0% frequency) and increased in frequency (98%–100%) at day 210 (ORF 1a: A510V, P141S, A446S, S301A; ORF 1b: L280F, R205C; S: P9L, LGVYY141Ydel, A701T; ORF 3a: L65F; and M: T7I). Of these, the Spike mutations were mapped to the N-terminal domain (LGVYY141Ydel) and an uncommon mutation downstream of the furin cleavage site (A701T) (Fig. 4).

Overall, non-lineage defining mutations were identified throughout the genome, with most mutations observed in ORF1a and ORF1b that constitute approximately two-thirds of the SARS-CoV-2 genome (Fig. 3); however, mutations were observed at the same frequency in ORF 1a and ORF 1b as in Spike (Fig. 4). These cases were typified by the emergence of non-lineage defining mutations predominantly in ORF 1ab and Spike and may affect viral replication, viral assembly, viral entry, and immune response modulation. These included the emergence of transient and fixed mutations, some of which may be host driven and confer temporary or long-lasting fitness advantage. The emergence of these mutations in these individual and other immunocompromised individuals in different reports throughout the pandemic may be indicative of convergent evolution and host adaptation.

## DISCUSSION

In this retrospective study, over 5,000 individuals were screened for repeat SARS-CoV-2 positive diagnoses. Of these 75, 72 were reinfections and 3 were persistent infections. Phylogenetic data were then linked to clinical metadata and confirmed that all persistent infections were in the three PLWH with very low CD4+ T cell counts (<30 cells/µL), and not in the seven PLWH with CD4+ T cell counts greater than 200 cells/µL and the three PLWH without CD4+ T cell count data, nor in the 23 individuals living without HIV, nor the 39 individuals without clinical metadata (Table S1). Increased viral evolution was identified in these individuals, especially in Spike, with most mutations associated with immune evasion.

Intra-host viral evolution in these cases of persistent infection was typified by emergence and accumulation of uncommon mutations and mutations particularly in Spike that have the potential to alter various aspects of viral replication, assembly, immune modulation, ACE2 binding, and cell fusion. Furthermore, we identified uncommon mutations with a low global prevalence at the time of diagnosis which, however, became prevalent in later VOCs with immune escape potential. SARS-CoV-2 diversity has expanded, and COVID-19 cases continue to fluctuate among global populations suggesting that the evolution trajectory and seasonality of SARS-CoV-2 remain unpredictable. Studying the evolution of SARS-CoV-2 in immunocompromised individuals is crucial to mapping the trajectory of SARS-CoV-2 evolution.

Reinfection with SARS-CoV-2 under endemic conditions is estimated to occur 487 days (range: 90–1,916 days) after maximum antibody response (27) and has been attributed to the depletion of specific antibodies (28, 29) or the acquisition of mutations in the virus that allows it to evade host immunity. In this work, reinfection with genetically distinct variants occurred at a median of 315.5 days (range: 62–692 days) after the initial infection (Fig. 1 and 2), which is largely in line with the progressive decline in SARS-CoV-2 immune-mediated protection that follows infection to levels that are insufficient for cross-protection to phylogenetically distinct variants. Waning immunity, coupled with viral-mediated immune escape of subsequent VOCs, most likely explains why all reinfections were ascribed to sequentially emerging variants including (Beta, Delta, and Omicron variants).

Robust humoral and T cell-mediated immune responses allow healthy adults to achieve SARS-CoV-2 clearance within 14 days (30), and in contrast, immunocompromised PLWH may be more susceptible to prolonged SARS-CoV-2 infection compared to healthy PLWH (CD4+ T cell ≥200 cells/µL) and those who are HIV negative (31). While studies investigating the evolutionary dynamics of SARS-CoV-2 focused predominantly on persistent infections in severely immunocompromised PLWH coupled with extensive viral intra-host evolution (2, 5, 7, 32, 33), persistent infections can also occur in healthy PLWH and HIV negative individuals. A large-scale community-based study, the United Kingdom (34), reported a population prevalence of 0.5% (381/70,000) of persistent infections, and these infections lasted between 30 and 60 days. Given that the immunological status of these individuals was not shared, it is unclear if these infections were associated with immunocompromised conditions. Additionally, in cases where paired, consecutive samples were available per participant, the corresponding sequences showed no evidence of viral evolution at the consensus level which may be suggestive of non-replicating viruses. In a comparative study, SARS-CoV-2 exhibited distinct evolutionary patterns and intra-host genetic diversity in diverse groups of PLWH and people who were HIV negative (10). Advanced HIV disease in severely immunocompromised individuals was associated with increased genetic diversity in Spike compared while immunocompetent PLWH and people who were HIV negative showed no significant difference in SARS-CoV-2 Spike diversity (10). The rapid intra-host diversification of Spike at critical functional sites in severely immunocompromised PLWH was linked to prolonged viral shedding and was typified by emergence of lineages prior to the detection and dissemination of these lineages in global populations. Additional studies investigating SARS-CoV-2 evolution in an immunologically diverse population of PLWH and people who are HIV negative may provide insight into community-level prevalence of persistent infections and the evolutionary trajectory of SARS-CoV-2 in immunologically diverse populations.

A recurring observation during persistent infection is accelerated viral evolution with the emergence of mutations due to host selection pressures in contrast to the limited intra-host diversity observed during acute infections (2, 3, 6, 32, 33, 35). The cases of SARS-CoV-2 persistence in immunocompromised individuals described in this study were typified by the emergence and disappearance of transient mutations, as well as fixation of other mutations throughout the genome (Fig. 3 and 4). The patterns of intra-host evolution may be attributed to interactions between viral replication and host-specific factors that drove the emergence and fixation of some mutations that favor immune evasion and infectivity resulting in host adaptation.

Although functional studies were not conducted to assess the implications of the mutations identified in this work, several of the mutations identified were characterized by others and are summarized in Table 2. Of interest was the emergence of mutations that were detected in later variants, including VOC (Table 2), particularly mutations detected in the N-terminal and receptor binding domains of Spike that influence ACE2 binding affinity, protein stability, replication, assembly, antigenicity, and immunogenicity. The N-terminal deletions (case 1: 143–144del; case 2: 244del; case 3: 141del, 243del; Fig. 3 and 4) facilitate immune escape by altering immunodominant epitopes that are targeted by neutralizing antibodies with some also improving viral infectivity (35, 36). Deletions at positions 143–144 and 242–247 are present in Alpha and Beta and Omicron variants (Table 2), respectively, and have been observed in several cases of SARS-CoV-2 persistence in different classes of immunocompromised individuals (10, 37, 38). The transient N-terminal domain mutations E96A and S254F could also influence aspects of antibody recognition. Similarly, non-lineage defining mutations accumulated in the receptor-binding domain (case 1: D420Y, Q493K, Q498K; case 2: S494P, P499R; case 3: E484K; Fig. 3, 4 and Table 2). Variations of the receptor-binding domain mutation D420N decrease protein stability, while Q493R increases protein stability and Q493L and Q498R decrease protein stability, respectively (COV2Var (wchscu.cn), Table 2). In addition to enhancing resistance to neutralizing antibodies (14), E484K, Q493R, and Q498R also enhance ACE2-binding affinity particularly in conjunction with N501Y (Table 2) (39); with Q493R reported to emerge during administration of therapeutic interventions (5, 40). Over the course of the infection, Q493R, and not Q498R, becomes fixed along with the lineage defining mutation N501Y and together they may facilitate cross-species transmission (Fig. 3 and 4). Variations of mutations at positions 493 and 498 have been noted in immunocompromised patients throughout the pandemic (38). Spike cleavage is required for virus entry followed by viral fusion with the host membrane. Thus, the emergence of mutations upstream and downstream of the Spike cleavage site (case 2: P681R, A701V; case 3: A701T, Fig. 4) could broaden host cell tropism and potentially enhance SARS-CoV-2 infectivity. These mutations or variations thereof were detected in subsequent VOCs including Beta, Delta, and Omicron variants (S: P681R, A701V; Table 2) and in immunocompromised individuals persistently infected with SARS-CoV-2 (38).

Despite rigorous surveillance in South Africa throughout the pandemic, similar Spike sequences identified in this work were not detected locally (case 1: Switzerland, EPI_ISL_981725; case 2: United States of America, OK245542.1, 2020; case 3: United States, OK245542.1, 2020; and Germany, EPI_ISL_1439701, 2021) suggesting no or minimal onward local spread of the mutations that evolved during the course of persistent infection. Similarly, the detection of uncommon mutations with low global transmission (Fig. 3) may be a consequence of random mutations that were subject to negative selection at the population level or adaptive intra-host evolution that may come at a fitness cost that hinders onward transmission. It does, however, support the hypothesis of the emergence and accumulation of novel mutations during persistent infection.

This work has several limitations. First, the lack of consecutive longitudinal samples limited the resolution of SARS-CoV-2 evolution during persistent infections. Second, the viruses identified during persistent infection were not isolated and subjected to neutralization and binding affinity assays to assess the impact of the mutation on viral fitness.

This work of persistent infection identified mutations that were seen in later VOCs. It provides valuable insights into the evolutionary trajectory of SARS-CoV-2 in immunocompromised individuals and specifically the emergence of variants with mutations that facilitate immune escape, threaten vaccine efficacies, and lead to potential fluxes in resurgence. Similar to other case reports and series (2, 5, 7, 32, 33), all individuals with persistent infections were in severely immunocompromised PLWH reported to be on antiretroviral treatment. However, CD4+ T cell counts were low which may be indicative of poor antiretroviral adherence, antiretroviral drug resistance, and/or co-infections/comorbidities that may impact treatment effectiveness and increase susceptibility to persistent infection. Further work is needed to determine the relationship between CD4+ T cell count and persistent infection. This study re-affirms the need for access and adherence to effective antiretroviral programs, ongoing surveillance of immunocompromised individuals.

## Data Availability

All genomics data are available in the NCBI Sequence Read Archive (https://www.ncbi.nlm.nih.gov/sra) and GISAID (https://gisaid.org/) using the accession numbers reported in Table 1.

## SUPPLEMENTAL MATERIAL

Figure S1 jvi.00780-25-s0001.tif Depth and distribution of sequencing reads for persistent SARS-CoV-2 infection, case 1.

Figure S2 jvi.00780-25-s0002.tif Depth and distribution of sequencing reads for persistent SARS-CoV-2 infection, case 2.

Figure S3 jvi.00780-25-s0003.tif Depth and distribution of sequencing reads for persistent SARS-CoV-2 infection, case 3.

Table S1 jvi.00780-25-s0004.xlsb General characteristics and clinical metadata of 75 paired cases of SARS-CoV-2 infection.

ASM does not own the copyrights to Supplemental Material that may be linked to, or accessed through, an article. The authors have granted ASM a non-exclusive, world-wide license to publish the Supplemental Material files. Please contact the corresponding author directly for reuse.
