# Supplementary figures and images for: Intra-host SARS-CoV-2 diversity in immunocompromised people living with HIV provides insight into the evolutionary trajectory of SARS-CoV-2
Source: J Virol. 2025 Sep 5;99(10):e00780-25. doi: 10.1128/jvi.00780-25 (PMC12548466; doi:10.1128/jvi.00780-25)

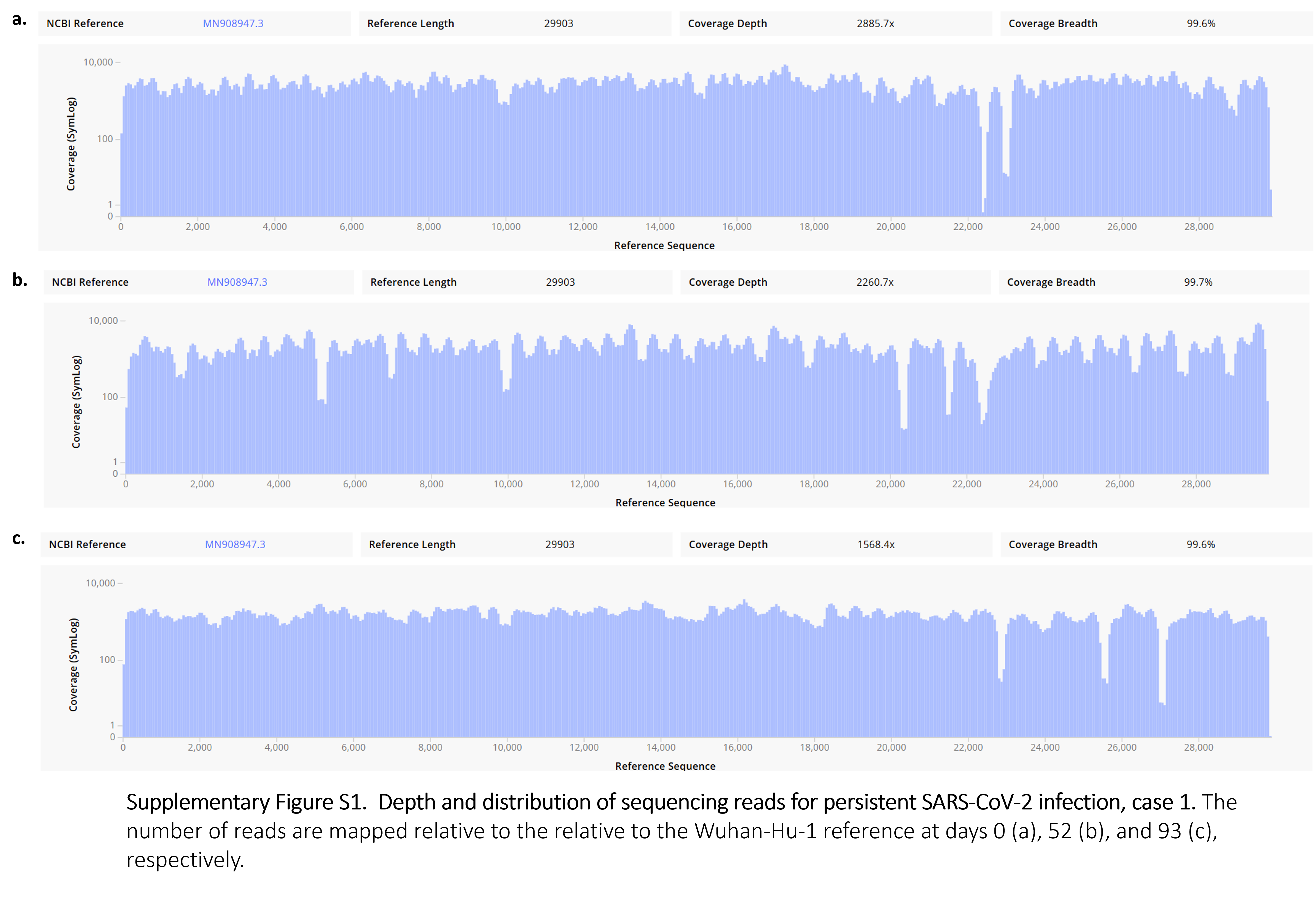

Supplement: Figure S1 — Depth and distribution of sequencing reads for persistent SARS-CoV-2 infection, case 1. [file jvi.00780-25-s0001.tif]

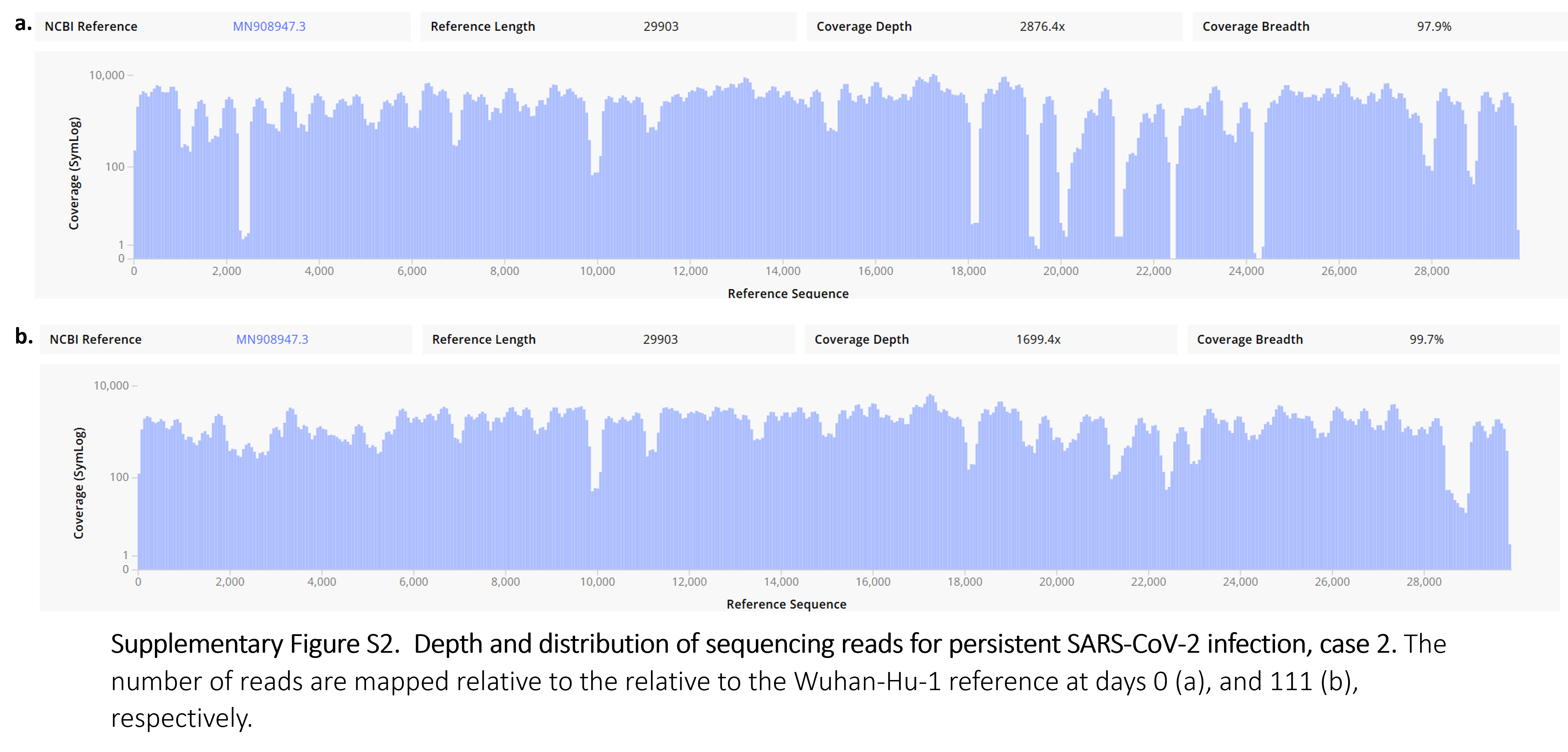

Supplement: Figure S2 — Depth and distribution of sequencing reads for persistent SARS-CoV-2 infection, case 2. [file jvi.00780-25-s0002.tif]

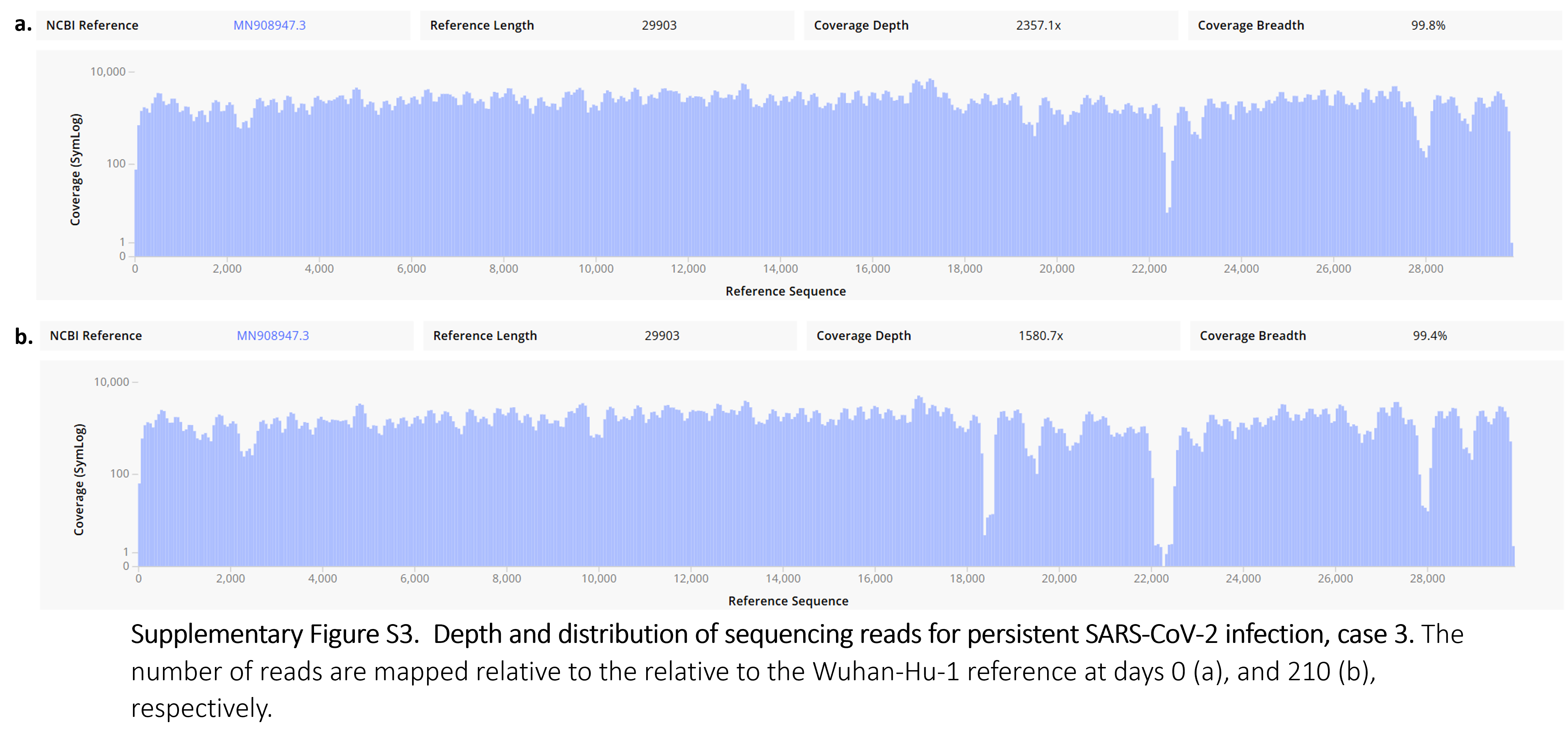

Supplement: Figure S3 — Depth and distribution of sequencing reads for persistent SARS-CoV-2 infection, case 3. [file jvi.00780-25-s0003.tif]
